# Mycosis Fungoides and Granulomatous Slack Skin: A Single Entity With Distinct Clinical Phenotypes

**DOI:** 10.1097/DAD.0000000000002590

**Published:** 2023-11-21

**Authors:** Farrah Bakr, Stela Ziaj, José Cardoso, E. Mary Wain, Jeffrey Theaker, Sean Whittaker, Fiona Child, Alistair Robson

**Affiliations:** * St John's Institute of Dermatology, Guy's and St Thomas' NHS Trust, London, United Kingdom;; † Department of Pathology, Southampton General Hospital, Southampton, United Kingdom; and; ‡ Instituto Português de Oncologia de Lisboa, Francisco Gentil, Lisboa, Portugal.

**Keywords:** primary cutaneous T-cell lymphoma, granulomatous mycosis fungoides, granulomatous slack skin

## Abstract

Granulomatous cutaneous T-cell lymphoma includes mycosis fungoides with significant granulomatous inflammation (GMF) and granulomatous slack skin (GSS), listed in the WHO classification as a subtype of mycosis fungoides (MFs).^1^ These overlapping entities have shared clinical and histopathologic features which can present a diagnostic challenge. The dominance of the granulomatous infiltrate and the often sparse lymphocytic infiltrate frequently with minimal cytological atypia are features that distract from the correct diagnosis, even when raised by the clinician. We describe the clinical and histopathologic characteristics of 3 cases of granulomatous cutaneous T-cell lymphoma, illustrate the close clinical and pathologic relationship between GMF and GSS and emphasize the diagnostic difficulties that the granulomatous infiltrate can present. Furthermore, we demonstrate, for the first time, considerable elastolysis in a significant proportion of classical (Alibert-Bazin) MF lesions and therefore postulate that the differences observed between GMF and GSS are one of degree and secondary to their anatomic location rather than reflecting meaningful separate entities.

## INTRODUCTION

Granulomatous mycosis fungoide (GMF) is a rare subtype of cutaneous T-cell lymphoma (CTCL) occurring in approximately 6% of all MF cases.^2^ Histologically arbitrarily defined as a granulomatous reaction that constitutes at least 25% of the infiltrate,^3^ most frequently with intimately admixed epithelioid granulomata, and multinucleated giant cells are often present.^2,4^ The granulomatous reaction can be extensive and obscure the associated malignant lymphoid population. Epidermotropism is only observed in half of the cases and cytological atypia is often minimal.^3^ Thus, many such cases are misdiagnosed as granulomatous dermatitis, with subsequent diagnostic delay.^5,6^ GMF lesions clinically present as classic (Alibert-Bazin) MF.^3–5,7^ In contrast, granulomatous slack skin (GSS) is clinically distinctive, with erythematous patches and plaques, often poikilodermatous that develop into pendulous folds of lax skin occurring at intertriginous regions.^8^ Despite this distinct clinical presentation, it has a similar histology to GMF; a granulomatous proliferation with variably heavy admixed T lymphocytes. Nevertheless, numerous multinucleated foreign body giant cells and loss of elastic fibers are hallmarks of GSS; however, elastophagocytosis is only directly observed in a minority.^3^ Although elastic tissue loss is also observed in GMF, it is reported to be more extensive in GSS lesions.^9^

In this study, we present 3 cases of granulomatous CTCL, which illustrate the histologic overlap between GMF and GSS, and also demonstrate often considerable dermal elastic tissue loss in classic (Alibert-Bazin) MF.

## CASES AND RESULTS

**FIGURE 1.**
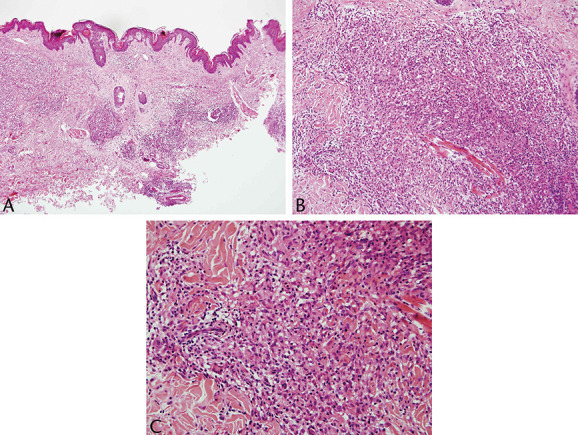
A–C, Diagnostic biopsy demonstrated a patchy nonepidermotropic dermal infiltrate dominated by granulomata and admixed small lymphocytes.

**FIGURE 2.**
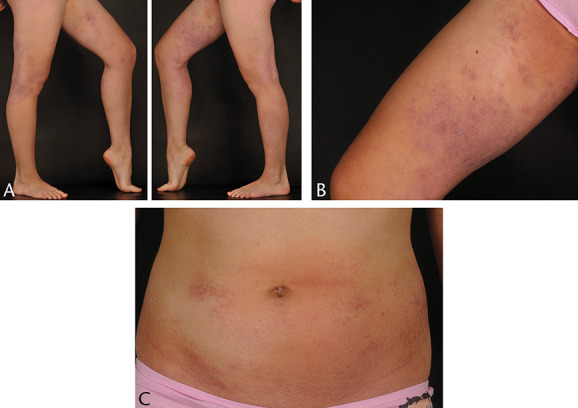
A–C, Widespread patches, plaques, and poikilodermatous foci (case 1).

**FIGURE 3.**
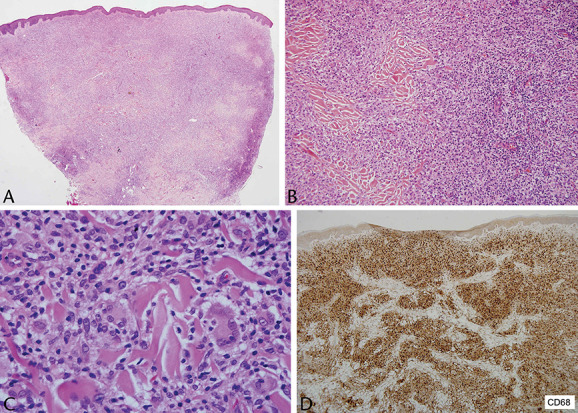
A–C, Diffuse dermal population of small lymphocytes and admixed macrophages, some multinucleate. D, Immunohistochemistry with CD68 highlights the numerous macrophages intimately admixed with the lymphocytic population.

**FIGURE 4.**
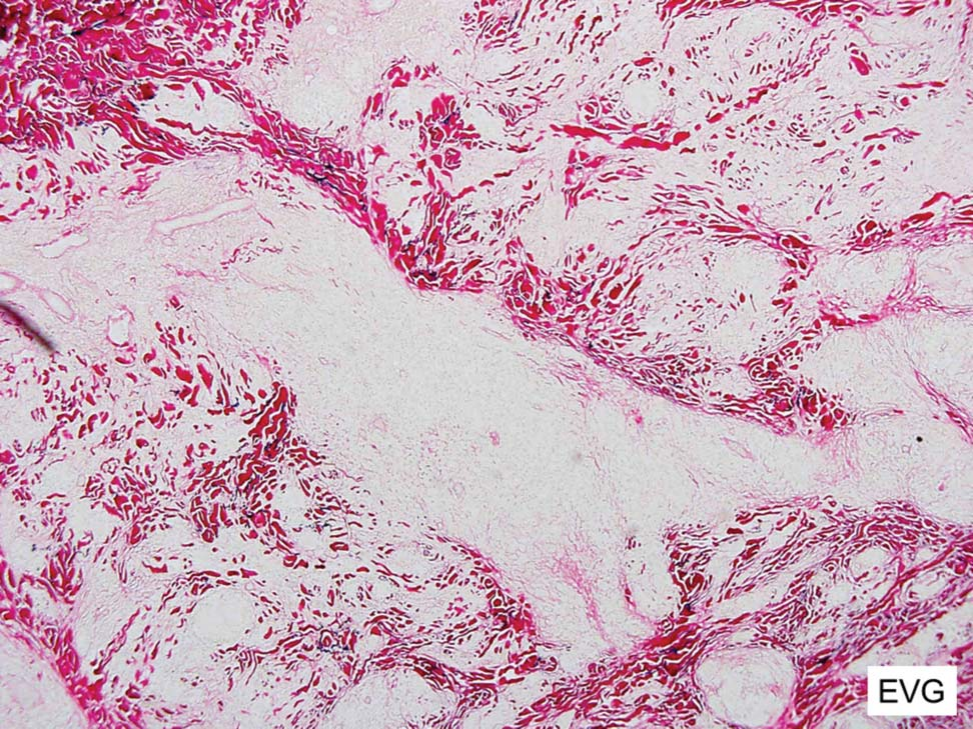
Elastic van Gieson demonstrates a diffuse loss of dermal elastic tissue.

**FIGURE 5.**
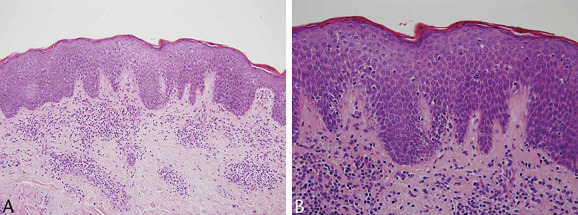
A, B, Case 1. A second biopsy taken from a clinical patch demonstrated a perivascular infiltrate with sparse epidermotropism. The lymphocytes have mild atypia.

Case 1: A 15-year-old girl with type 5 skin presented with a 3-year history of cutaneous lesions, which had started as a small violaceous patch on her right inner thigh. She subsequently developed further lesions on the left thigh, calves, abdomen, and arms. An initial skin biopsy demonstrated a patchy superficial and deep dermal granulomatous infiltrate with moderate numbers of admixed small lymphocytes (Figs. 1A–C); this had been interpreted as an unusual form of sarcoidosis, pending exclusion of infection. Because of slow but continuous extension of the cutaneous lesions, the patient was referred to our institute for further management. On examination, the patient had ill-defined violaceous patches with foci of poikiloderma on the inner aspect of both thighs with small plaques. Scaly erythematous patches were present on the calves and abdomen. On the lateral aspect of both arms, there were ill-defined macular hypopigmented areas with perifollicular erythema (Figs. 2A–C). There was no palpable lymphadenopathy or hepatosplenomegaly. A repeat biopsy from a plaque entirely consisted of a dense diffuse nonepidermotropic infiltrate extending throughout the dermis, comprising macrophages and lymphocytes. The macrophages formed a diffuse syncytium rather than discrete granulomata. Lymphocyte atypia was minimal (Figs. 3A–C). Elastic Van Gieson stain revealed an almost complete loss of elastic fibers (Fig. 4). A second biopsy from a patch revealed a mild superficial dermal lymphocytic infiltrate with limited extension of small lymphocytes into a nonspongiotic epidermis. Cytological atypia was mild, but some of the lymphocytes in the epidermis exhibited cerebriform nuclei with perinuclear halos; no Pautrier microabscesses were identified (Figs. 5A, B). Immunohistochemistry of both infiltrates demonstrated the predominant population to have a CD4^+^ CD45RA + T-cell phenotype with loss of CD7. In the first biopsy, CD68 confirmed the dominant macrophage component (Fig. 3D). Molecular analysis of T-cell receptor gene rearrangements revealed an identical clone in both biopsies. The constellation of clinical, histologic, immunophenotypical, and molecular studies indicated a diagnosis of granulomatous mycosis fungoides (GMF), stage IB: Further investigations, including full blood count, liver and kidney functions tests, erythrocyte sedimentation rate, lymphocyte subsets, Sezary cell count, and HTLV-1 serology were normal.

**FIGURE 6.**
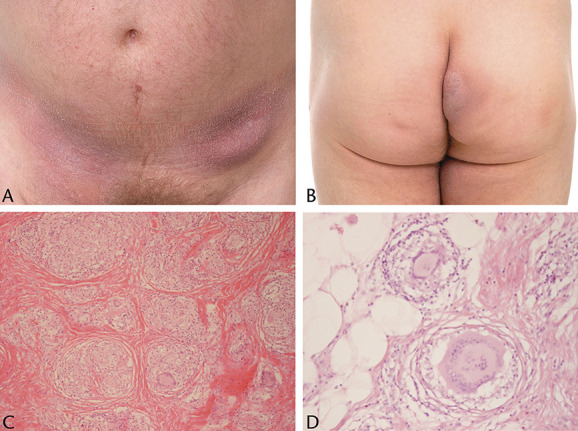
A, B, Indurated plaques of the lower abdomen and an early pendulous lesion of buttock skin. C, D, Widespread multinucleate foreign body-type giant cells and only sparse lymphocytes, the latter without appreciable atypia (case 2).

Case 2: A 50-year-old gentleman presented with a one-year history of a purplish nodular plaque on his right upper thigh, which had gradually increased in size over this period. An incisional biopsy revealed multiple non-necrotising granulomata, characterized by prominent and confluent multinucleate foreign body-type giant cells, with only sparsely admixed small lymphocytes. There was no epidermotropism or significant cytological atypia; an initial diagnosis of atypical sarcoidosis was suggested. He continued to develop more plaques in the groin, lower abdomen, and buttocks (Figs. 6A, B). Over the next 2 years, he developed a large area of cutaneous laxity in the right groin. The excess skin was surgically excised and again the histology was felt to be in keeping with cutaneous sarcoidosis albeit atypical. The patient continued to develop new areas of slack skin that commenced as thickened, violaceous, and minimally scaly plaques limited to his groins, lower abdomen, and buttocks, which evolved into pendulous folds of skin. He had normal chest radiographs, pulmonary function tests and full blood count, serum angiotensin-converting enzyme, and calcium levels throughout the course of his disease. Subsequently, a further biopsy was taken and revealed multiple non-necrotizing epithelioid granulomata with strikingly prominent giant cells and a sparse population of small lymphocytes (Figs. 6C, D). No foreign material was identified. Ziehl-Neelsen and fungal stains were negative. Elastic van Gieson histochemistry confirmed widespread loss of elastin. PCR performed on a fresh skin biopsy demonstrated a clonal population of T cells. A diagnosis of GSS was made.

**FIGURE 7.**
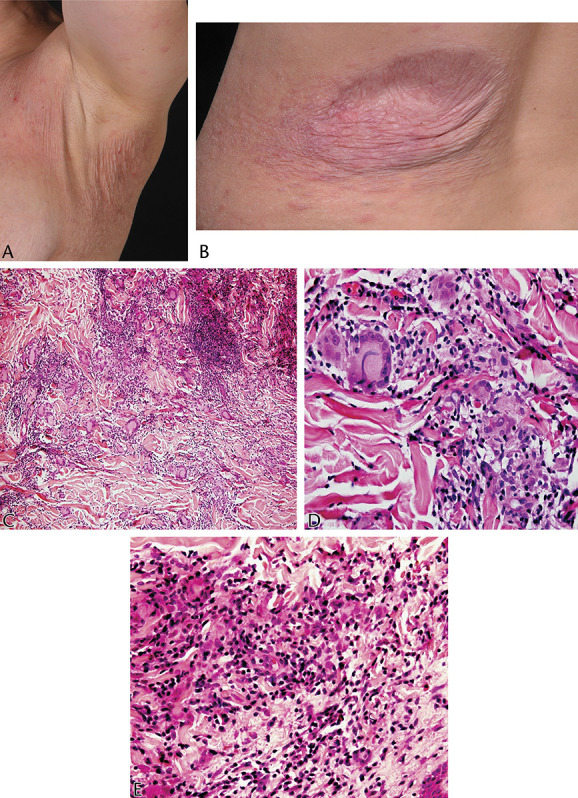
A, B, Wrinkling, atrophy, and laxity of an erythematous patch affecting the axilla. The adjacent skin has subtle patches and a few papules (case 3). C–E.

Case 3: A 44-year-old woman presented with a 6-year history of skin disease, which had begun as small patches of pruritus on her back. There was no response to antifungal or topical steroids and she subsequently developed areas of atrophy, initially attributed to the steroids. Further areas of slack skin, preceded by erythema and scaling evolved (Figs. 7A, B) and she was referred to our center. On examination, she was found to have folds of slack skin with atrophy affecting the left lower back, left axilla, and right inner thigh. She had scaly erythematous areas in the right axilla and on the right chest wall. Further examination revealed shotty, inguinal lymph nodes, but no palpable axillary, cervical lymphadenopathy, or organomegaly. A biopsy taken from an affected area on her back consisted of a dense interstitial and nodular lymphoid cell infiltrate, having focal epidermotropism, which was composed of small- and medium-sized lymphoid cells, most of which had only mild cytological atypia. There were abundant admixed multinucleate giant cells; a giant cell with elastophagocytosis was noted (Figs. 7C–E). Immunohistochemistry confirmed most cells in the infiltrate to be CD8(+) T lymphocytes, without loss of T-cell-associated antigens. The morphologic features were those of GMF. A CT/PET showed no evidence of systemic disease.

**FIGURE 8.**
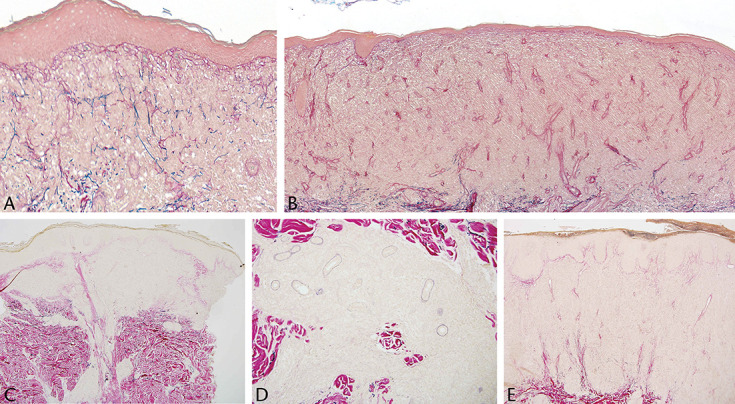
A–E, Six images of marked absence of elastic tissue within biopsies from lesions of classic Alibert Bazin MF (Elastic van Gieson).

**FIGURE 9.**
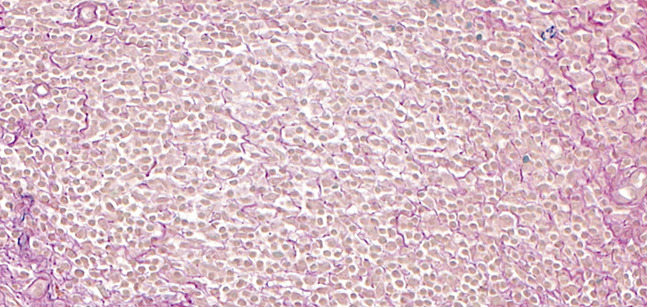
Closer view of almost complete absence with only a few small, fragmented fibers (Elastic van Gieson).

**FIGURE 10.**
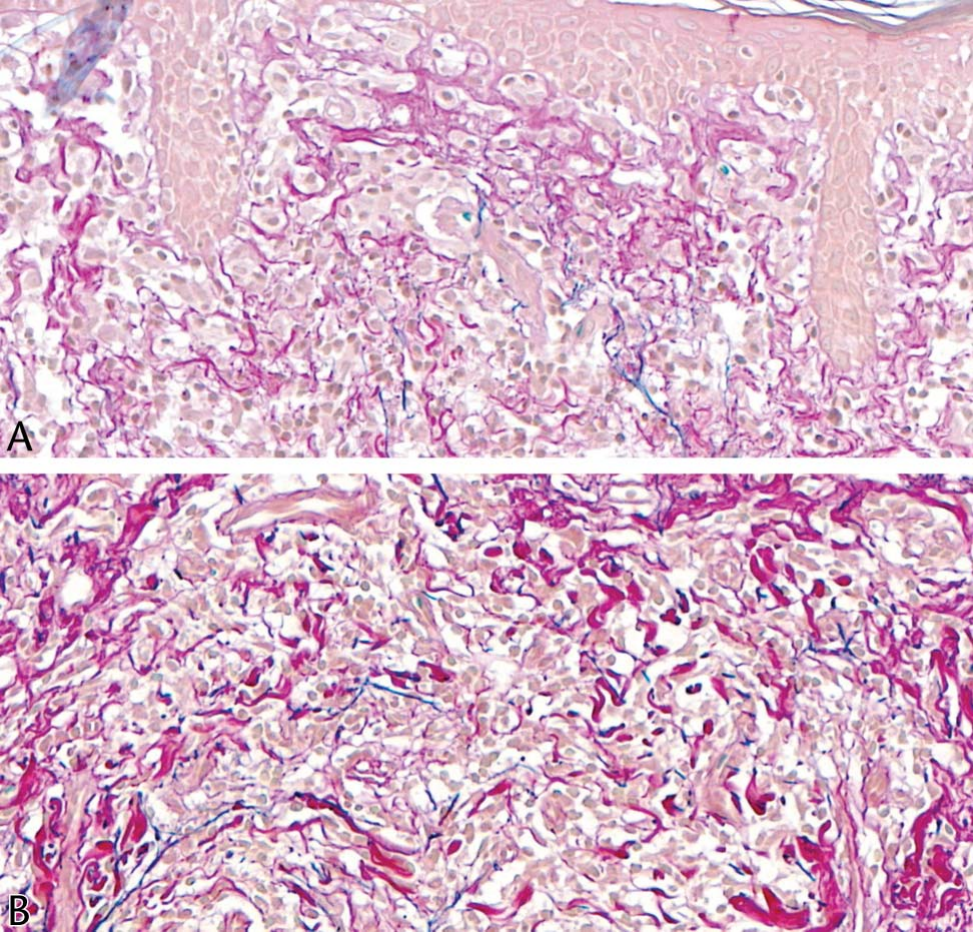
A, B, Preserved elastic fibers irregularly distributed within the infiltrate (Elastic van Gieson).

Twenty-one biopsies from 20 patients with histologically confirmed classic (Alibert-Bazin) MF were analyzed with elastic van Gieson histochemistry for assessment of dermal elastic tissue. None originated from the axilla or groin. Elastic van Gieson staining confirmed substantial loss of dermal elastic tissue in lesional samples in 16 patients, without the clinical formation of lax skin folds (Figs. 8 and 9). Of the remainder, 3 had possible focal loss and the other 2 seemed to have retained elastic tissue (Fig. 10). No granulomata or elastophagocytosis were identified on H&E review. Furthermore, there was no evidence that elastic tissue had been merely displaced downward into the deeper dermis in classic Alibert-Bazin MF or cases of granulomatous MF and GSS described above.

## DISCUSSION

Granuloma formation is well-documented in systemic lymphomas,^10^ but uncommonly observed in primary cutaneous lymphomas.^6^ Partly in view of their rarity, the clinicopathologic features of granulomatous CTCLs are often not recognized. Furthermore, in GMF and GSS, the dermal infiltrate may be dominated by granulomas rather than lymphocytes, often with a lack of epidermotropism and little cytological atypia of the lymphocytes, features that do not readily suggest a diagnosis of cutaneous T-cell lymphoma and often leading pathologists to favor inflammatory and infectious conditions. Moreover, in cases that present clinically with skin laxity or folds, the clinician may not propose lymphoma as a candidate diagnosis.

The 3 cases presented herein highlight the similarities between GMF and GSS. In cases 2 and 3, there were clinical areas of skin laxity or folds, 1 of which (case 3) had more prevalent areas of patches of MF. Case 2 was dominated by multinucleate—foreign body type—giant cells, whereas case 3 had a predominantly lymphocytic dermal population and scattered admixed giant cells, with some foci of sparse epidermotropism; it was this case that had the only observable elastophagocytosis. Case 1 presented with clinical features of MF, but the diagnosis was delayed histologically because of the dominance of epithelioid granulomata and/or a more diffuse population of individual macrophages, some multinucleate, with admixed lymphocytes having little atypia. Indeed, lymphocyte cytological atypia was mild in all cases. Case 2 was notable for the striking dominance of foreign-body type giant cells and very few lymphocytes, perhaps an indication of a late stage in the disease process, supported by this patient having the longest prediagnosis clinical period.

In 2008, the EORTC published the results of a workshop analyzing 15 cases of GMF and 2 of GSS and made some of these observations.^3^ In particular, no specific histologic feature was uniquely associated with GMF or GSS. The mechanism for bulky pendulous lesions in GSS is believed to be secondary to a reduction in dermal elastic tissue and the presence of multinucleate giant cells a response to this. Interestingly, however, elastophagocytosis was directly observed in only 1 of our cases, despite multiple biopsies and numerous granulomata, and was also observed only rarely in the EORTC series. The apparent close relationship between GSS and GMF raised the question of elastic tissue abnormalities as a feature of MF generally. Our finding that most biopsies from Alibert-Bazin MF lesions also have significant absence of elastic fibers underlines this feature as a common finding in these diseases, and simultaneously suggests that the granuloma formation itself is unlikely to have a pathogenic role. It can be postulated, therefore, that the GMF and GSS differ from classical MF in the extent of this process, and GMF and GSS depending on whether the marked and widespread elastolysis affects gravity-sensitive sites. The basis for this presumed elastolysis is unclear. Metalloproteinases are enzymes that play a central role in tissue remodeling including the digestion of elastic and collagenous tissues. Although much work has been performed characterizing the process in the invasion and metastasis of carcinomas,^11^ there are as yet no mechanistic studies in cutaneous lymphoma. Because most patients with MF, GMF, and GSS do not succumb to advanced and metastatic disease, the fact that dissolution of elastic tissue seems a widespread observation, implies that elastolysis is not an indicator of aggressive disease and imminent dissemination.

**FIGURE 11.**
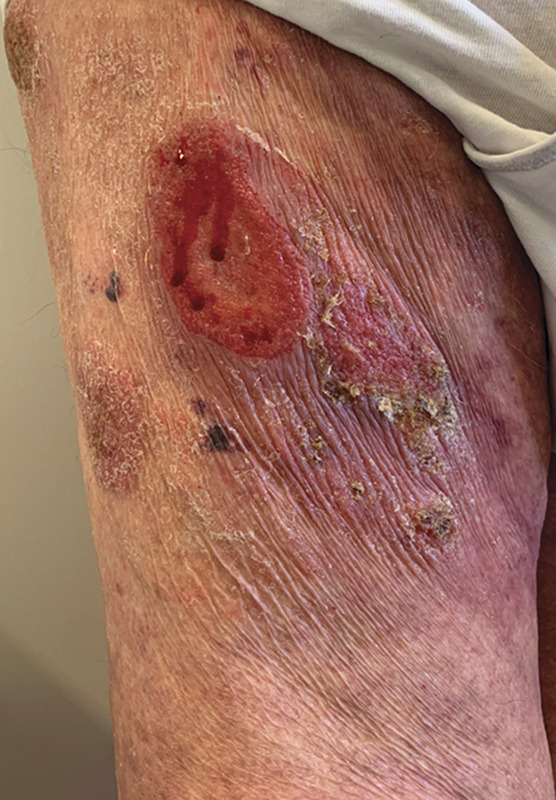
Conspicuous wrinkling affecting an ulcerated plaque in a lesion of classical MF (C/o Dr Mariana Cravo, Department of Dermatology, IPOLFG, Lisboa, Portugal).

There is evidence for elastic fiber dermal network abnormalities in a range of nongranulomatous forms of CTCL. Anetodermic MF, an entity distinct from GSS, also histologically consists of a predominant lymphocytic infiltrate with admixed histiocytes, giant cells, and a complete loss of elastic fibers in the dermis.^12^ Unlike GSS, the lesions do not affect the flexural areas and elastophagocytosis is rarely observed. Poikilodermatous MF is characterized by wrinkled skin lesions with a “cigarette paper”-like appearance along with atrophy, reticulate pigmentation, telangiectasia, and scaling. Histologically, a perivascular lymphohistiocytic infiltrate is present in the dermis with evidence of ectatic vessels and pigmentary incontinence. Unlike granulomatous CTCL, epidermotropism is often observed and a loss of elastic fibers in the superficial dermis is documented.^13^ Cutis-laxa-like MF, which is characterized by large areas of wrinkled skin on nonflexural sites is histologically comprised of an interstitial infiltrate of atypical lymphocytes and histiocytes within collagen bundles. Unlike in granulomatous CTCL, the cerebriform lymphocytes exhibit epidermotropism with focal loss and fragmentation of elastic fibers in the absence of elastophagocytosis.^14^ Finally, the clinical observation of skin wrinkling in patches of classical MF is commonplace (Fig. 11). This may be a manifestation of the widespread elastolysis that we have demonstrated. We do not know whether the observed elastolysis is unique to neoplastic lymphocytic infiltrates or whether it could potentially serve as a diagnostic aid in difficult cases. This may be a fruitful area for further investigation.

## CONCLUSIONS

The loss of elastic tissue observed in all these MF variants, including classic (Alibert-Bazin) MF, could clinically result in wrinkling or laxity of the skin. However, because there are no significant reproducible histologic differences between GMF and GSS, we propose that the distinction between the GMF and GSS mirrors the degree of elastolysis, response to the elastic fragmentation, and—most significantly—the anatomic location of the disease, rather than these representing distinct clinicopathologic entities.
